# Upright activity and higher motor function may preserve bone mineral density within 6 months of stroke: a longitudinal study

**DOI:** 10.1007/s11657-017-0414-4

**Published:** 2018-01-08

**Authors:** Karen Borschmann, Sandra Iuliano, Ali Ghasem-Zadeh, Leonid Churilov, Marco Y. C. Pang, Julie Bernhardt

**Affiliations:** 1 School of Health Science, La Trobe University, Bundoora, Australia; 2 The Florey Institute of Neuroscience and Mental Health, University of Melbourne, Heidelberg, Australia; 3 Department of Medicine, Austin Health, University of Melbourne, Heidelberg, Australia; 4 Department of Endocrinology, Austin Health, University of Melbourne, Heidelberg, Australia; 5 Department of Rehabilitation Sciences, Hong Kong Polytechnic University, Hung Hom, Hong Kong

**Keywords:** Stroke, Bone loss, Bone mineral density, Microstructure, HR-pQCT, Physical activity

## Abstract

**Purpose:**

Bone fragility contributes to increased fracture risk, but little is known about the emergence of post-stroke bone loss. We investigated skeletal changes and relationships with physical activity, stroke severity, motor control and lean mass within 6 months of stroke.

**Methods:**

This is a prospective observational study. Participants were non-diabetic but unable to walk within 2 weeks of first stroke. Distal tibial volumetric bone mineral density (vBMD, *primary outcome*), bone geometry and microstructure (high-resolution peripheral quantitative computed tomography) were assessed at baseline and 6 months, as were secondary outcomes total body bone mineral content and lean mass (dual energy X-ray absorptiometry), bone metabolism (serum osteocalcin, N-terminal propeptide of type 1 procollagen (P1NP), C-terminal telopeptide of type 1 collagen (CTX)), physical activity (PAL2 accelerometer) and motor control (Chedoke McMaster) which were also measured at 1 and 3 months.

**Results:**

Thirty-seven participants (69.7 years (SD 11.6), 37.8% females, NIHSS 12.6 (SD 4.7)) were included. The magnitude of difference in vBMD between paretic and non-paretic legs increased within 6 months, with a greater reduction observed in paretic legs (mean difference = 1.5% (95% CI 0.5, 2.6), *p* = 0.007). At 6 months, better motor control was associated with less bone loss since stroke (*r* = 0.46, *p* = 0.02). A trend towards less bone loss was observed in people who regained independent walking compared to those who did not (*p* = 0.053). Higher baseline daily count of standing up was associated with less change in bone turnover over 6 months: osteocalcin (*r* = −0.51, *p* = 0.01), P1NP (*r* = −0.47, p = 0.01), CTX (*r* = −0.53, p = 0.01).

**Conclusion:**

Better motor control and walking recovery were associated with reduced bone loss. Interventions targeting these impairments from early post-stroke are warranted.

**Clinical trial registration:**

URL: http://www.anzctr.org.au. Unique identifier: ACTRN12612000123842.

**Electronic supplementary material:**

The online version of this article (10.1007/s11657-017-0414-4) contains supplementary material, which is available to authorized users.

## Introduction

Although age-standardised hip fracture rates vary globally from < 10 to nearly 500/100,000 population [1], increased fracture risk is recognised as a common and serious stroke sequela [2–4]. The likelihood of hip fracture at 12 months post-stroke, particularly in paretic legs, increases two to four times to that of age- and sex-matched controls [2, 5]. Compared to people without stroke, people who fracture after stroke have higher mortality at 1 month (3.3 vs 8.8%, *p* = 0.008) [6] are less likely to regain the ability to walk by hospital discharge (69.2 vs 38.1%, *p <* 0.001) [6] and are more likely to be discharged to residential care (OR 2.69 (95% CI 1.11, 6.54) *p* = 0.028) [3].

Bone mineral density (BMD), a surrogate measure of bone strength, is thought to decline post-stroke due to increased bone resorption and reduced formation, consistent with uncoupling of bone remodelling [7]. While bone loss and increased fracture risk in people with chronic stroke (> 12 months after onset) are well recognised, bone loss likely occurs early after stroke [7]. However, evidence on the timing of post-stroke bone loss, and the contributions of age, gender and stroke impairments, is limited by lack of longitudinal data. Consequently, the magnitude of change that is expected to occur from early after stroke is unknown, and little is known about factors that may influence post-stroke bone loss.

Most studies of post-stroke bone loss have utilised dual energy X-ray absorptiometry (DXA) to estimate areal BMD (g/cm^2^) of the paretic side hip. Reduced aBMD has been associated with the length of time people are unable to walk [8], and reductions in muscle strength [9] and mass [10]. However, due to the inherent error in accuracy and reproducibility of DXA imaging, particularly due to difficulty with consistent positioning of people with stroke [9], and the limitations of projecting 3-dimensional structures onto 2-dimensions, areal BMD lacks sensitivity and specificity for fracture prediction [11]. High-resolution peripheral quantitative computed tomography (HR-pQCT, voxel size 82 μm) provides estimation of volumetric BMD (vBMD, mgHA/cm^3^) and bone microstructure, which are better determinants of bone strength than areal BMD [12], and only scan slices that are common between assessments are included in analyses, which assumes that the bone area is constant over time. Furthermore, trabecular and cortical bone, each of which contributes differently to bone strength, can be quantified separately [11].

To our knowledge, no reports exist of HR-pQCT use to longitudinally evaluate lower limb bone loss from early after stroke. Low-resolution pQCT (voxel size 500 μm) was recently used to examine changes over 12 months in bone structure and density of lower limbs of people who were more than 12 months post-stroke [13]: trabecular bone density at the distal tibia (4% site of bone length) reduced by 1.8% (SD 0.6, *p* = 0.006) in the 20 people who completed the study.

In the present study, we utilised HR-pQCT to determine the magnitude of skeletal changes between 2 weeks and 6 months of first stroke. We hypothesised that total vBMD of the distal tibia would reduce more in paretic compared to non-paretic legs. Moreover, we hypothesised that less vBMD change in paretic legs would be associated with better motor function, and with regaining the ability to walk within 6 months of stroke.

## Methods

The full protocol for this prospective observational study has been published [14]; details are provided here in brief. Adults > 40 years admitted to one of two public hospitals in Melbourne (Australia) within 1 week of first stroke, diagnosed with hemispheric stroke, medically stable, unable to walk 10 ft independently and able to follow simple commands were eligible. Exclusion criteria were the following: previous stroke, diabetes, other neurological disease, other condition limiting function (e.g., limb amputation) or use of bone-specific medication (e.g., bisphosphonates). Informed consent was obtained from all participants included in the study.

## Outcome measurement

Assessments were undertaken at baseline (within 2 weeks of onset), 1 month post-baseline and 3 and 6 months post-stroke. Demographic and stroke-related data were collected from medical records at baseline. At each assessment, participants reported any falls that they had experienced since the previous assessment. Study-related adverse events were collected, defined as any study-related event that required medical or nursing attention. HR-pQCT scans were undertaken at baseline and 6 months; all other assessments were completed at each time point.

### Primary outcome

The primary outcome (change in the magnitude of difference of total vBMD between paretic and non-paretic legs at the distal tibia between baseline and 6 months) was measured at 7% of bone length from the distal end using HR-pQCT (Xtreme CT, Scanco Medical AG, Switzerland. Coefficient of variation (CV, measurement precision) was 1.3% [15]. This is a region that contains both trabecular and cortical bone. Tibial bone strength has been associated with both vertebral fracture (OR 2.92, 95% CI 1.14, 3.03) and non-vertebral fracture (OR = 2.64, 95% CI 1.63, 4.27), and it is therefore considered that properties of the tibia are reasonably representative of other bone sites (Vilayphiou et al. 2010).

### Secondary outcomes

Cortical and trabecular vBMD (mgHA/cm^3^), cortical thickness (mm), cortical and trabecular cross-sectional area (mm^2^) and total, cortical and trabecular bone mass (g) at the distal tibiae were also measured. Trabecular bone volume fraction was calculated (bone volume/total volume), and trabecular number (TbN, 1/mm), thickness (TbTh, μm), separation (TbSp, μm) and inhomogeneity of trabecular network (distribution of separation, TbSp SD, μm) were calculated based on the manufacturer’s algorithm. CV of density measurements was 0.6–1.4% and structural parameters was 0.9–4.4% [15]. The clinically relevant difference (i.e. least significant change [16]) is calculated √2 × 1.65 × CV; density measures = 1.4–3.3% and structural measures = 2.1–10.3%. Lean mass and bone mineral content of the total body and legs were determined from total body DXA scans (DPX-L, version 1.3z: Lunar Corp., Madison, USA).

Physical activity was measured using PAL2 accelerometer (Gorman ProMed Pty Ltd., Australia) [17] to register the number of changes in position and the amount of time spent lying, sitting, standing and walking. The device was worn for 1 day from 8am to 5pm at each time point [18].

Disability was classified using the Modified Rankin Scale [19]. Motor function of the paretic leg was evaluated with Chedoke McMaster Stroke Assessment [20]. Quadriceps muscle strength was assessed seated using hand-held dynamometry (Nicholas Manual Muscle Tester; Lafayette Instruments, Lafayette, USA); the average of three test scores were standardised to body mass [21]. Walking ability was classified on Functional Ambulation Classification [22].

Fasting serum markers of bone turnover were assessed (osteocalcin, resorption marker serum C-terminal telopeptide of types 1 collagen (CTX) and formation marker N-terminal propeptide of type 1 procollagen (P1NP)) by electrochemiluminenescence immunoassay (Elecsys 1010 Analytics, Roche Diagnostics, Germany, intra- and inter-assay CV 3–8%) [23].

## Sample size calculation

Using the difference between paretic and non-paretic limbs in 6-month change of distal tibia vBMD from pilot data (*n* = 10, mean reduction paretic = − 1.43% (SD 1.92), non-paretic = − 0.45% (SD 1.37)), and assuming one sample, two-tailed test with criterion for significance of *p* = 0.05, 25 participants would provide 80% power to detect a significant effect of this magnitude or larger. Adjusting for an expected mortality of 30% [24], 33 participants were required for the main study.

## Statistical analysis

People who did not complete the trial due to death or withdrawal (*n* = 4) were not included in any analyses due to their data being not missing at random [25]. The complete case analysis was conducted on the combined pilot and main study datasets for all outcomes.

The primary outcome (change in the difference in vBMD between paretic and non-paretic legs over 6 months) was compared to zero using a one-sample *t* test. Secondary outcomes: the association between the change in vBMD and paretic leg motor function at 6 months was assessed using Spearman’s correlation coefficient, while the relationship between change in vBMD and regaining the ability to walk by 6 months (not able to walk = FAC (Functional Ambulation Classification) 1–4 vs able to walk = FAC 5–6) was investigated using Wilcoxon rank-sum test.

Distributions of secondary outcomes (bone turnover markers, lean mass and bone mineral content) were tested by Shapiro-Wilk test, then log transformed as required. Exploratory analyses of change in secondary outcomes over 6 months were undertaken by generalised estimating equation. Change in secondary HR-pQCT measures was examined as described for the primary outcome. Analyses were performed using STATA v13IC software (StataCorp, College Station, USA). Due to exploratory nature of this research, a significance level of 0.05 was set for all analyses. Separate sub-group analyses (e.g. age, sex, ethnicity) were not undertaken due to small sample.

## Results

**Fig. 1:**
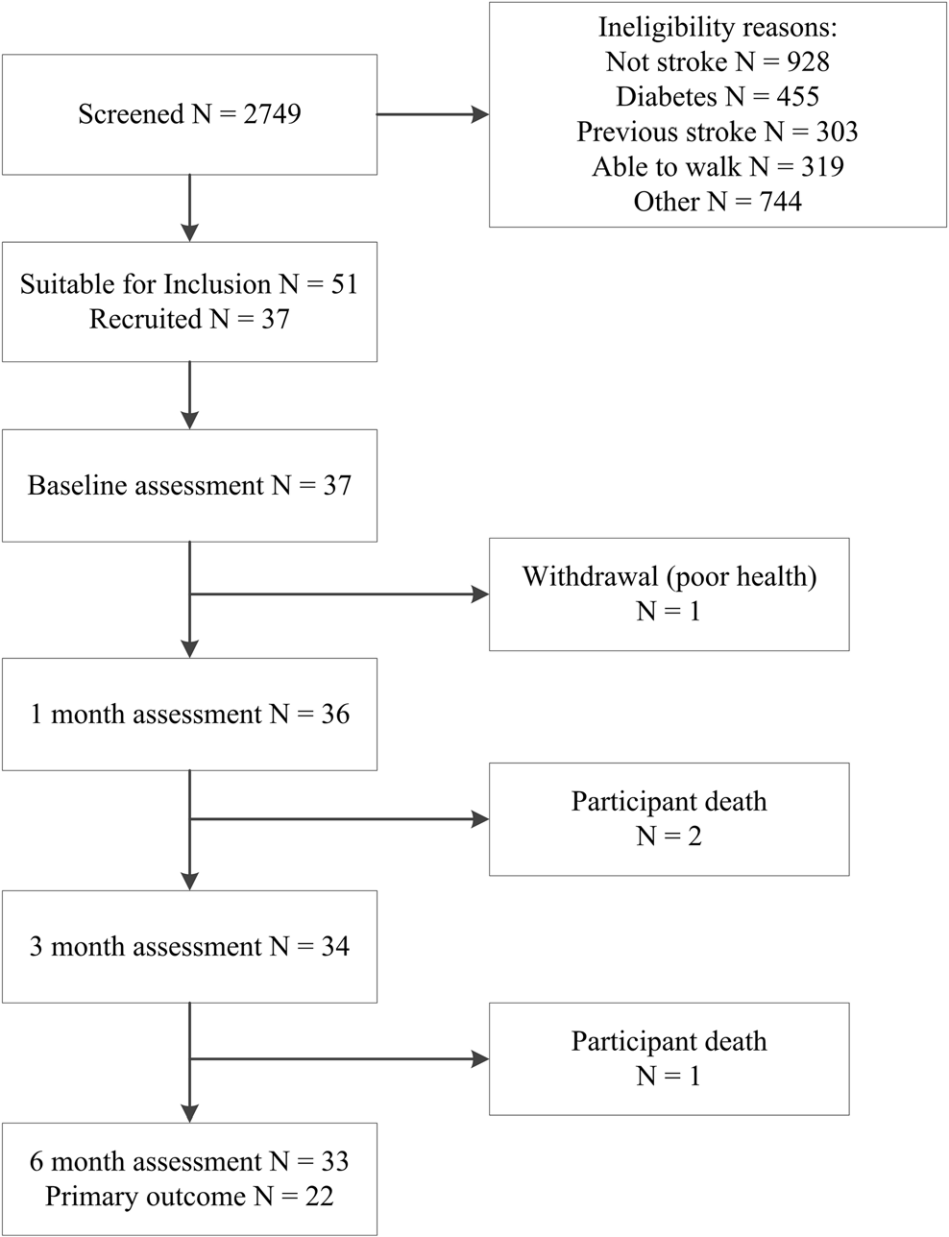
Participant recruitment and retention

In total, 2749 patients were screened and 37 provided consent (Table 1). Reasons for exclusion and participant flow through the study are shown in Fig. 1. Participants who completed the study (*n* = 33) had proportionally fewer haemorrhagic strokes compared to those who did not (*n* = 4), (*χ*^2^ = 9.4, *p* = 0.02). No study-related adverse events were reported. Twenty-two people (67%) fell at least once during the course of the study. The median number of falls was 2 (IQR 1, 3), and two people were fractured (1 hip, 1 wrist). Of those who completed the study, 22 (67%) had adequate bone images for primary outcome analysis. Reasons for missing data were the following: movement during scan (*n* = 5), declined or unavailable (*n* = 3), illness (*n* = 2) and scanner not available (*n* = 1). Participants who provided primary outcome data were younger than those who did not (66.0 ± 12.1 years vs 75.3 ± 8.3, *p =* 0.03).

**Table 1:** Baseline demographics and stroke characteristics

Characteristic	All recruits	Completed trial			
			Primary outcome analysis		
			Yes	No	p*
	*n* = 37	*n* = 33	*n* = 22	*n* = 11	
Sex, female	14 (37.8)	13 (39.4)	10 (45.5)	3 (27.3)	0.31
Age, mean (SD)	69.7 (11.6)	69.1 (11.7)	66.0 (12.1)	75.3 (8.3)	0.03
Lived with others prior to stroke	28 (75.7)	25 (75.8)	17 (77.3)	8 (72.7)	0.77
Employed	14 (37.8)	13 (39.4)	10 (45.5)	3 (27.3)	0.32
Primary language not English	7 (18.9)	7 (21.2)	4 (18.2)	3 (27.3)	0.55
Walked prior, no gait aid	32 (86.5)	29 (87.9)	20 (90.9)	9 (81.8)	0.45
BMI, mean (SD)	27.1 (5.4)	27.5 (5.4)	27.3 (5.2)	27.8(6.4)	0.41
Number comorbidities	4.2 (2.9)	4.0 (2.7)	4.2 (3.1)	3.6 (1.9)	0.72
Past medical history					
Previous fracture	12 (32.4)	11 (33.3)	7 (31.8)	4 (36.4)	0.79
Hypertension	18 (48.7)	15 (45.5)	9 (40.9)	6 (54.5)	0.46
High cholesterol	12 (32.4)	10 (30.3)	6 (27.3)	4 (36.4)	0.59
Ischaemic heart disease	7 (18.9)	5 (15.2)	4 (18.2)	1 (9.1)	0.49
Atrial fibrillation	7 (18.9)	6 (18.2)	3 (13.6)	3 (27.3)	0.34
Musculoskeletal	15 (40.5)	14 (42.4)	8 (36.4)	6 (54.5)	0.32
Smoking history					
Current smoker	12 (32.4)	10 (30.3)	6 (27.3)	4 (36.4)	
Never smoked	14 (37.8)	12 (36.4)	9 (40.9)	3 (27.3)	
Previous smoker	11 (29.7)	11 (29.7)	7 (31.8)	4 (36.4)	
Vit D/calcium supplementation	7 (18.9)	7 (21.2)	5 (22.7)	2 (12.9)	0.76
Stroke severity†	12.6 (4.7)	12.9 (4.8)	12.6 (4.8)	13.5 (5.0)	
Group mean (SD)	5 (13.5)	4 (12.1)	4 (18.2)	0	0.30
Mild, *n* (%)	24 (64.9)	21 (63.6)	13 (59.1)	8 (72.7)	
Moderate, *n* (%)	8 (21.6)	8 (24.2)	5 (22.7)	3 (27.3)	
Severe, *n* (%)					
Stroke classification‡					0.67
TACI	18 (48.6)	18 (54.6)	11 (50.0)	7 (63.6)	
PACI	11 (29.7)	10 (30.3)	7 (31.8)	3 (27.3)	
LACI	4 (10.8)	3 (9.1)	3 (13.6)	0	
Haemorrhage	4 (10.8)	2 (6.1)	1 (4.5)	1 (9.1)	

*n* (%) unless stated otherwise

*BMI*, body mass index, kg/m^2^; *TACI*/*PACI*, total/partial anterior circulation infarct; *LACI*, lacunar circulation infarct; *Vit D*, vitamin D

*Participants who completed the study, comparison of those who did and did not have primary outcome data: Wilcoxon rank-sums or Fisher’s Exact Test

†NIHSS (National Institutes of Health Stroke Scale): mild < 8, moderate 8–16, severe > 16

‡Significant difference (*p* = 0.02) between participants who did and did not complete study

### HR-pQCT-derived parameters

At baseline, total vBMD of the paretic leg was 268.4 mgHA/cm^3^ (SD 68.4) and non-paretic leg was 272.0 mgHA/cm^3^ (SD 67.4) (Table 2). By 6 months, vBMD reduced by 2.4% (SD 2.7, *p* = 0.001) in paretic legs, but did not change in non-paretic legs. The between-limb difference was not significant at either time point. Confirming the primary hypothesis, the magnitude of change in the between-limb difference in vBMD increased by 1.53% (95% CI 0.46, 2.61, *p* = 0.01) within 6 months. Similarly, significant changes were observed across 6 months for the between-leg difference in total and cortical bone mass (*p* = 0.01, 0.004, respectively), and cortical and trabecular areas (*p* = 0.02, 0.006, respectively) (Table 2). All HR-pQCT-derived variables are contained in the supplemental table.

**Table 2:** HR-pQCT-derived total volumetric bone mineral density, cortical thickness and bone area of distal tibiae within 2 weeks of stroke and 6-month change (*n* = 22)

	Baseline			6-month % change		
	Paretic	Non-par	BW-leg diff %	Paretic	Non-par	BW-leg diff % mean (95% CI)*
Total vBMD, mgHA/cm^3^	268.39 (68.41)	272.59 (67.37)	2.15 (5.77)	− 2.4 (2.7)	− 1.0 (2.4)	1.53 (0.46, 2.61) *p* = 0.01
Total bone mass, g	186.2 (52.1)	190.0 (48.7)	3.0 (5.1)	− 2.5 (2.8)	− 1.0 (2.4)	1.56 (0.47, 2.65) *p =* 0.01
Cortical bone mass, g	76.6 (34.7)	75.9 (33.2)	0.2 (12.7)	− 8.5 (10.4)	− 4.2 (7.0) 5.26 (1.91, 8.62) *p =* 0.004	
Trabecular bone mass, g	100.1 (28.8)	104.8 (28.1)	6.2 (9.4)	− 0.4 (1.8)	− 1.2 (2.2)	− 0.84 (− 1.84, 0.17) *p =* 0.10
Cortical area, mm^2^	108.28 (42.52)	107.69 (40.74)	0.28 (10.59)	− 7.0 (8.6)	− 3.1 (5.9)	4.42 (1.78, 7.06) *p* = 0.002
Trabecular area, mm^2^	691.24 (132.38)	699.51 (136.36)	1.14 (4.39)	0.5 (0.09)	− 0.02 (− 0.79)	− 0.58 (− 0.97, − 0.19) *p* = 0.006

Mean (SD)

*Non-par*, non-paretic; *vBMD*, volumetric bone mineral density; *BW-leg Diff*, between-leg difference = [(non-paretic–paretic)/paretic] × 100

*One sample *t* test

### DXA derived parameters

At baseline, 21 participants (72.4%) had normal BMD [26], seven (24.1%) were osteopenic (1–2.5 SD below reference range) and one person was osteoporotic (≥ 2.5 SD below reference). Significant changes in bone mineral content were not observed until the 6-month assessment, at which time reductions were observed for total body (2.8%, IQR 0.6, 5.6, *p* = 0.001) and paretic legs (3.4%, IQR 0.5, 6.1, *p* < 0.001), Table 3. Change in lean mass could not be estimated via generalised estimating equation as estimates diverged (correlation > 1); Wilcoxon signed-rank test of baseline and 6-month data showed that lean mass did not change significantly.

**Table 3:** Bone turnover markers and DXA-derived bone mineral content and lean mass at baseline and 6 months after stroke

	Baseline *N* = 30 BTM/29 DXA	6 months *N* = 28	6-month % change *N* = 26	Significance*
CTX, pg/ml	568.75 (361.2, 735.6)	597.6 (385.55, 847)	4.89 (− 28.01, 45.65)	0.55
P1NP, μg/l	38.45 (26.38, 43.0)	76.17 (51.99, 97.84)	94.42 (64.63, 171.13)	< 0.001
Osteocalcin ng/ml	15.07 (11.18, 20.0)	22.26 (15.61, 29.81)	42.71 (− 0.65, 100.4)	< 0.001
Muscle mass, kg				
Total body	47.00 (40.44, 52.73)	48.20 (40.33, 54.63)	1.2% (− 3.2, 4.2)	0.49
Paretic leg	7.47 (6.58, 8.36)	7.46 (6.69, 8.48)	0.4% (− 3.4, 5.1)	0.82
Non-paretic	7.22 (5.88, 8.45)	7.55 (6.29, 8.66)	1.3% (− 2.8, 6.8)	0.09
BMC, g				
Total body	2919 (2135, 3244)	2705 (2091, 3095)	− 2.8% (− 5.6, − 0.6)	0.039
Paretic leg	533 (439, 603)	512 (423, 585)	− 3.4% (− 6.1, − 0.5)	< 0.001
Non-paretic leg	536 (415, 601)	528 (426, 600)	− 0.3% (− 2.1, 0.3)	0.19

Data presented median (IQR); *BMC*, bone mineral content (grams); *BTM*, bone turnover markers (resorption); *CTX*, carboxyterminal crosslinked telopeptide of type 1 collagen; formation, *P1NP*, N-terminal propeptide of type 1 procollagen

*Generalised estimating equation for all variables except lean mass and non-paretic leg BMC due to estimates diverging (correlation > 1), so assessed by Wilcoxon signed-rank test

### Bone turnover markers

Compared to baseline results, P1NP was elevated at every time point, and osteocalcin was elevated by 3 months; both remained elevated at 6 months, Table 3. No significant changes were detected in CTX. By 1 month, P1NP increased by 59.3% (IQR 12.2, 104.5, *p* = 0.001) which is more than its least significant change (19.9%), and at 3 months, osteocalcin was 29.7% (IQR 8.4, 74.4, *p* = 0.001) higher than baseline.

### Physical assessments

**Fig. 2:**
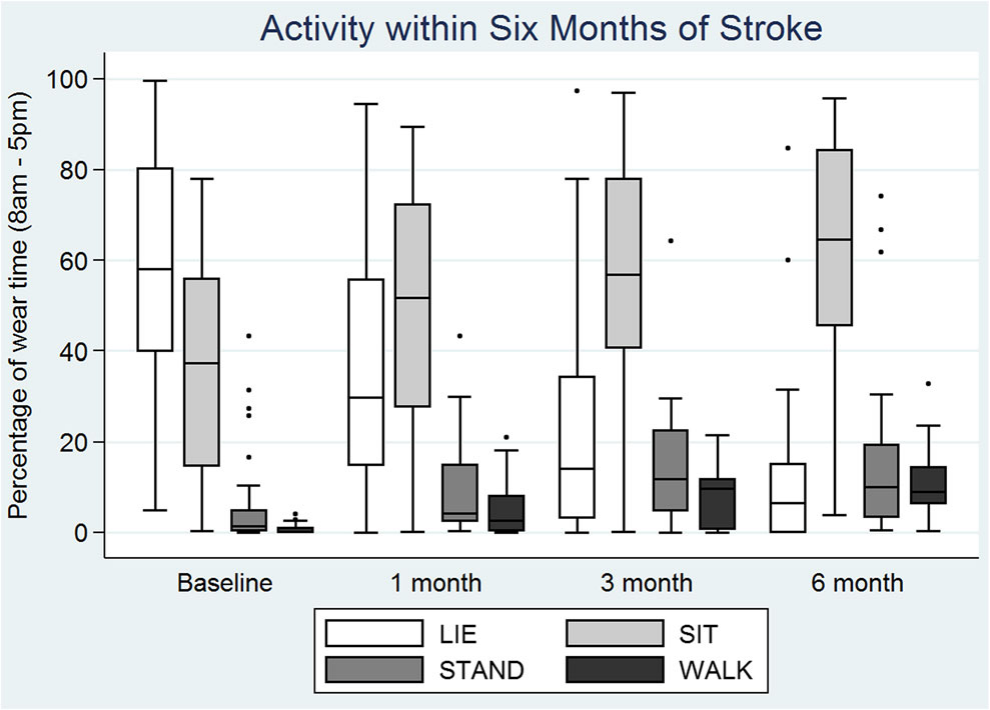
Physical activity between 2 weeks and 6 months of stroke

Independent walking was achieved by 67% of participants (*n* = 22, FAC > 4, Fig. 2), by 54.5 (IQR 27.8, 98.0) days post-stroke. At baseline, participants lay in bed for 57.8% (IQR 40.9, 79.0) of the day, and spent < 2% of time upright, standing (1.4%, IQR 0.3, 5.2) or walking (0.2%, IQR 0.0, 1.6) (Fig. 2). Participants stood up 14 (IQR 6, 28) times in a day. At 6 months, participants sat for 62.5% (IQR 39.9, 82.8) of the day, stood for 12.5% (IQR 2.9, 21.1), walked for 8.7% (IQR 6.1, 15.1) and stood up 53 times (IQR 38, 73), all *p* < 0.01.

Paretic leg motor function was reduced at baseline (2.5, IQR 2, 4) and improved but remained below normal (< 7/7) (5, IQR 3, 6, *p* < 0.001). Quadriceps strength at baseline was 66.0% higher (IQR 11.5, 107.7, *p* < 0.001) in non-paretic legs than paretic legs. Strength increased in both legs during the study; at 6 months, non-paretic legs were 18.9% (IQR 4.3, 44.4, *p* < 0.001) stronger than paretic legs.

As hypothesised, change in vBMD of paretic legs was significantly associated with leg motor function at 6 months (*p* = 0.02), Table 4. The association between change in paretic leg vBMD and regaining the ability to walk independently approached significance (*p* = 0.053): − 1.51% (IQR − 3.67, − 0.09) in those who regained walking compared to − 5.54% (IQR − 6.25, − 3.63) in those who did not. Higher stroke severity, reduced motor function of the paretic leg and lower physical activity at baseline were associated with changes in bone turnover markers but not with change in vBMD.

**Table 4:** Associations between 6-month changes in vBMD of paretic leg and bone turnover markers, stroke severity, physical activity and paretic leg motor control

	% change vBMD paretic leg	% change P1NP	% change osteocalcin	% change CTX
Baseline: NIHSS	− 0.04	0.46	0.31	0.52
	0.85	0.01	0.08	0.001
	24	28	28	28
Baseline: daily count of standing up	0.17	− 0.47	− 0.51	− 0.53
	0.44	0.01	0.01	0.01
	23	27	27	27
Baseline: proportion of time sedentary	− 0.08	0.54	0.43	0.28
	0.72	0.003	0.02	0.16
	23	27	27	27
Baseline: paretic-leg motor function†	0.31	− 0.59	− 0.58	− 0.67
	0.16	0.001	0.001	0.0001
	23	28	28	28
6 month: paretic-leg motor function†	0.46	− 0.29	− 0.38	− 0.32
	0.02	0.14	0.047	0.10
	24	28	28	28

Cells contain Spearman’s rho, *p*, n.

*CTX*, C-terminal telopeptide of type 1 collagen; *NIHSS*, National Institute of Health Stroke Scale; *P1NP*, N-terminal propeptide of type 1 procollagen; *vBMD*, volumetric bone mineral density

*Significant at 0.05

†Chedoke McMaster Stroke Assessment

## Discussion

This was the first longitudinal examination of skeletal changes from early post-stroke using HR-pQCT. We observed an increase in the magnitude of difference in total vBMD between paretic and non-paretic legs, with a greater reduction in paretic legs. As expected [27], given the average age of approximately 70 years, the loss was evident predominantly in cortical rather than trabecular bone. Significant changes were observed in both cortical (− 7.0%, SD 8.6) and trabecular bone areas (0.5%, SD 0.09), which is consistent with cortical thinning and the concomitant increase in trabecular area. Bone loss exceeded the machine error margin of 1.4–3.3% and that expected due to ageing alone (0.5–1% in 12 months) [28]. Our observation of 2.4% (SD 2.8) reduction of vBMD in the paretic leg within 6 months is greater than the 1.4% (SD 0.3) reduction previously observed over 12 months in people who were 12–24 months post-stroke [13]. As expected, two thirds of participants fell at least once during the study [29], and two participants (6.1%) fractured within 6 months, in comparison to the annual Australian fracture rate of 0.18% (175/100,000 population) [30]. Combined, these data suggest that bone loss is likely highest within the first 6 months post-stroke, so prevention of bone loss during this rapid phase is critical to prevent fracture risk in this vulnerable patient population.

Our data showed that higher motor control at 6 months after stroke and regaining the ability to walk were associated with less reduction in vBMD of paretic legs. Baseline measures of higher stroke severity, lower paretic leg motor control and lower physical activity were associated with greater changes to bone metabolism markers but not changes in vBMD. The number of times that participants stood up, but not the proportion of time spent on their feet at baseline, was associated with 6-month change in bone resorption (CTX). This likely reflects bone response to discrete loads rather than length of loading, as bone cells desensitise to prolonged loading [36]. Results infer that regular weight bearing within the first week of stroke, and targeting leg motor control and reattainment of walking, may protect bone mass after stroke. This needs to be tested.

Similar to a previous longitudinal study of musculoskeletal changes using DXA from early after stroke [31], we did not observe changes in lean mass within 6 months of stroke. Evidence from longer-term longitudinal studies [8, 9] and a systematic review of predominantly cross-sectional chronic stroke data [32] suggest that loss of lean mass may occur after 6 months post-stroke, and the time taken to regain independent walking may mediate changes in both lean mass and BMD in paretic and non-paretic legs.

Previous reports of increased bone resorption in people with chronic stroke [7, 33, 34] are in contrast with our observation that bone resorption marker CTX did not change in the first 6 months of stroke. However, given that bone formation (P1NP) and turnover marker osteocalcin did increase, and studies of young healthy adults show that bone resorption occurs within 2 days of bed rest [35], it is likely that CTX increased early after stroke prior to baseline assessments. Interestingly, our observation of increased bone formation markers at 1 month that remained elevated at 6 months was in contrast with results from Poole (2009) who observed lower histological indices of bone formation in 14 people with moderate physical impairment (71 years (SD 11)) at 10 (SD 2) weeks post-stroke compared to controls.

A study limitation was the proportion of participants not included in the primary outcome (15/37, 41%), and the younger age of those who finished the study, so results may not be generalisable to older stroke survivors. Although movement during scanning is common, the large number of failed scans due to movement artefact may have influenced precision in some parameters [37]; however, clinically and statistically significant differences were observed in results. Due to the expected burden on participants who had high levels of impairment early after stroke, HR-pQCT scans were only assessed at baseline and 6-month follow-up. DXA scans, which only require subjects to lie still, were assessed at all time points. Given that DXA-derived changes in bone mineral content (BMC) were not observed in our participants until 6 months, and that participants could tolerate HR-pQCT testing of lower limbs early after stroke, HR-pQCT assessments within 6 months of stroke are warranted in future studies to examine the rate of change in bone microstructure.

### Summary

This was the first longitudinal examination of HR-pQCT-derived vBMD and microarchitecture, bone metabolism, lean mass and physical activity within 6 months of moderately severe stroke. Greatest bone loss was observed in cortical bone of paretic legs, beyond that expected from ageing alone. Recovery of walking and higher motor control were associated with less bone loss; therefore, targeting these impairments from early after stroke is warranted to establish interventions to reduce post-stroke bone loss.

## Electronic supplementary material

Supplemental Table 1 (DOCX 24 kb)
