# Supplementary material for: Upright activity and higher motor function may preserve bone mineral density within 6 months of stroke: a longitudinal study
Source: Arch Osteoporos. 2018 Jan 8;13(1):5. doi: 10.1007/s11657-017-0414-4 (PMC5758649; doi:10.1007/s11657-017-0414-4)
Supplement: Supplementary file 1 — (DOCX 24 kb) [file 11657_2017_414_MOESM1_ESM.docx]

ONLINE SUPPLEMENT.

Table I. All HR-pQCT Derived Bone Density and Structural Variables of Distal Tibiae within 2-weeks and at 6-months after Stroke (n=22)

|  | Baseline | | | Six-months | | | Six-month % change | | |
| --- | --- | --- | --- | --- | --- | --- | --- | --- | --- |
|  | Paretic | Non-par | BW-leg Diff % | Paretic | Non-par | BW-leg Diff % | Paretic | Non-par | BW-leg Diff % Mean (95% CI)^1^ |
| Total vBMD | 268.39  (68.41) | 272.59  (67.37) | 2.15  (5.77) | 262.37  (69.89) | 270.25  (68.83) | 3.69  (5.84) | -2.4  (2.7) | -1.0  (2.4) | 1.53  (0.46, 2.61)  *p*=0.01 |
| Cortical vBMD | 806.31  (77.47) | 803.63  (75.37) | -0.27  (2.70) | 791.46  (85.81) | 794.84  (80.06) | 0.55  (2.90) | -1.9  (2.7) | -1.2  (2.0) | 0.82  (-0.25, 1.89)  *p*=0.13 |
| Trabecular vBMD | 169.96  (42.50) | 176.66  (41.90) | 5.03  (8.51) | 168.36  (42.65) | 174.85  (42.56) | 4.80  (8.12) | -1.0  (1.8) | -1.13  (2.2) | 1.94  (-0.36, 4.23)  *p=*0.09 |
| Total bone mass | 186.2 (52.1) | 190.0 (48.7) | 3.0 (5.1) | 181.9 (52.4) | 188.4 (49.9) | 4.6 (5.5) | -2.5 (2.8) | -1.0 (2.4) | 1.56 (0.47, 2.65) *p=*0.01 |
| Cortical bone mass | 76.6 (34.7) | 75.9 (33.2) | 0.2 (12.7) | 71.8 (36.1) | 74.1 (34.9) | 5.5 (13.4) | -8.5 (10.4) | -4.2 (7.0) | 5.26 (1.91, 8.62) *p=*0.004 |
| Trabecular bone mass | 100.1 (28.8) | 104.8 (28.1) | 6.2 (9.4) | 99.6 (28.6) | 103.7 (28.5) | 5.4 (9.0) | -0.4 (1.8) | -1.2 (2.2) | -0.84 (-1.84, 0.17) *p=*0.10 |
| Cortical thickness | 0.96  (0.37) | 0.95  (0.36) | -0.17  (11.3) | 0.90  (0.39) | 0.93  (0.38) | 4.24  (11.85) | -7.1  (8.7) | -3.2  (6.2) | 3.70  (-1.88, 9.28)  *p*=0.18 |
| Cortical area | 108.28  (42.52) | 107.69  (40.74) | 0.28  (10.59) | 102.5  (44.59) | 105.78  (43.14) | 4.70  (11.27) | -7.0  (8.6) | -3.1  (5.9) | 9.5  (1.87, 17.14)  *p*=0.02 |
| Trabecular area | 691.24  (132.38) | 699.51  (136.36) | 1.14  (4.39) | 695.10  (133.49) | 699.38  (136.49) | 0.57  (4.24) | 0.5  (0.09) | -0.02  (-0.79) | -0.58  (-0.97, -0.19)  *p*=0.01 |
| Trabecular bone volume fraction | 14.16  (3.54) | 14.71  (3.50) | 5.01  (8.57) | 14.03  (3.55) | 14.58  (3.54) | 4.84  (8.14) | -1.0  (1.7) | -1.13  (2.2) | -0.84  (-4.10, 2.42)  *p*=0.60 |
| Number of trabeculae | 1.65  (0.36) | 1.71  (0.35) | 5.68  (10.39) | 1.66  (0.38) | 1.68  (0.36) | 2.66  (7.90) | 0.005  (0.06) | -2.1  (6.5) | 2.94  (-2.10,7.97)  *p*=0.24 |
| Trabecular thickness | 0.087  (0.014) | 0.085  (0.014) | -0.28  (6.98) | 0.086  (0.014) | 0.087  (0.014) | 2.22  (6.31) | -1.07  (6.6) | 1.3  (5.9) | 7.37  (2.14, 12.60)  *p*=0.01 |
| Trabecular separation | 0.57  (0.24) | 0.53  (0.16) | -5.14  (9.62) | 0.57  (0.24) | 0.54  (0.19) | -2.79  (7.65) | 0.04  (6.2) | 2.8  (6.9) | -3.07  (-8.37, 2.22)  *p*=0.24 |
| Inhomogeneity of trabecular network | 0.32  (0.27) | 0.29  (0.17) | -4.81  (13.46) | 0.32  (0.26) | 0.30  (0.22) | -3.37  (8.56) | 1.4  (8.3) | 3.7  (9.5) | 1.44  (-2.30, 5.17)  *p*=0.43 |

*Note*. mean (SD) or (95% CI).

BW-leg Diff=between-leg difference=[(non-paretic– paretic)/paretic]x100; ^1^ One sample t-test.

Bone mass=g; Cortical area=mm^2^; Cortical thickness=mm; Cortical periosteal perimeter=mm; Inhomogeneity of trabecular network=mm; Number of trabeculae=1/mm; Trabecular area=mm^2^; Trabecular bone volume fraction=bone volume/total volume, BV/TV; Trabecular separation=mm; Trabecular thickness=mm; vBMD=volumetric bone mineral density=mg HA/cm^3^
